# Trends of underweight, overweight, and obesity among older adults in China from 2008 to 2018: a national observational survey

**DOI:** 10.1186/s12889-023-16310-6

**Published:** 2023-07-18

**Authors:** Dina Jiesisibieke, Yuting Feng, Zhu Liduzi Jiesisibieke, Jue Liu, Liyuan Tao

**Affiliations:** 1 Research Center of Clinical Epidemiology, Peking University Third Hospital, Beijing, 100191 China; 2 Peking University Health Science Center, Beijing, 100191 China; 3 School of Traditional Chinese Medicine, Beijing University of Chinese Medicine, Beijing, 100029 China; 4 School of Public Health, The University of Hong Kong Li Ka Shing Faculty of Medicine, Hong Kong, Hong Kong; 5 Department of Epidemiology and Biostatistics, School of Public Health, Peking University, Beijing, 100191 China; 6 Key Laboratory of Epidemiology of Major Diseases (Peking University), Ministry of Education, Beijing, 100191 China

**Keywords:** Underweight, Overweight, Obesity, Older adults, Observational study

## Abstract

**Objective:**

This study aims to investigate the 10-year trends and disparities in underweight, overweight, and obesity among older adults aged 65 years and older in China from 2008 to 2018.

**Methods:**

We used four waves (2008, 2011, 2014, and 2018) of data from the Chinese Longitudinal Healthy Longevity Survey (CLHLS), a national community-based cross-sectional survey conducted every 2–3 years. Body weight and height were measured by trained assessors following standardized procedures. BMI was calculated and divided into underweight (< 18.5 kg/m^2^), normal (18.5–24.9 kg/m^2^), overweight (25.0-29.9 kg/m^2^), obese (≥ 30.0 kg/m^2^) according to WHO reference. Multinomial logistic regression models were used to examine factors related with abnormal BMI groups, after adjusting for potential confounders.

**Results:**

Among 46,543 older adults in China, the prevalence rates of underweight decreased with each survey year from 2008 to 2018, declining from 20.05 to 7.87% (p < 0.001). In contrast, the prevalence rates of overweight and obesity showed an increasing trend (all p < 0.001). Specifically, the prevalence of overweight rose from 12.82% to 2008 to 28.45% in 2018, and the prevalence of obesity increased from 1.62% to 2008 to 4.95% in 2018. In the multinomial logistic regression model, survey year, gender, residence, marital status, economic status, numbers of chronic diseases, smoking status, sleep quality, and functional disability were factors related with obesity.

**Conclusion:**

The prevalence rates of overweight and obesity were increasing while the prevalence of underweight and normal weight significantly decreased from 2008 to 2018 among older adults in China, which poses a huge challenge for chronic disease. There is an urgent need for intervention policy planning and early prevention of abnormal body weight for the preparation of an aging society.

**Supplementary Information:**

The online version contains supplementary material available at 10.1186/s12889-023-16310-6.

## Background

Overweight and obesity represent significant global public health concerns [1]. The prevalence of obesity worldwide has nearly tripled over the past approximately 50 years, reaching alarming levels. This poses a significant threat to public health as it is closely associated with various leading causes of morbidity and mortality, including coronary heart disease, cancers, hypertension, and type 2 diabetes mellitus [1–3]. A recent study combined conventional age-period projections with the concept of a wave-shaped obesity epidemic to predict the long-term prevalence of obesity in 18 European countries and the United States. The study indicated that obesity prevalence is expected to peak between 2026 and 2054, with the USA and UK reaching the highest maximum levels initially [4]. Although the USA and Europe currently have the highest obesity prevalence rates, obesity has also emerged as a significant public health issue in China, leading to substantial national healthcare expenditures [5].

Previous studies have demonstrated that average body weight steadily increases with age and reaches its peak between the ages of 50 to 65 years [2, 6]. However, the results of different countries were quite different [6, 7]. Considerable attention should be paid to the study of body weight issues among older adults because of the accelerated aging progress across the world [8]. As the most important developing country with the largest number of elderly people in the world, China is also confronted with the challenges of population aging [9]. The national prevalence estimates for overweight and obesity in adults aged ≥ 18 years old in China for the period 2015-19 were 34.3% and 16.4%, respectively, and the prevalence varied based on sex, age group, and geographical location [5]. Findings from the China Chronic Disease and Risk Factors Surveillance program, which was established in 2004, showed that the obesity prevalence among participants aged 18–69 years increased from 3.1% to 2004 to 8.1% in 2018 [10]. However, there is insufficient evidence focusing on trends in overweight and obesity prevalence among older adults in China. The Chinese Longitudinal Healthy Longevity Survey (CLHLS), which is another national tracking study focusing on the health status of older adults in China, provides us with the opportunity to better address the issues of underweight, overweight, and obesity among older adults [11].

In this study, we aimed to investigate the trends and disparities in body weight change among older adults aged 65 years and older in China from 2008 to 2018 and provide some information for more targeted policies, using the national cross-sectional data from eight representative health surveys among older adults conducted in China in 2008, 2011, 2014, and 2018.

## Methods

### Study population and data source

This is a national observational study using data from the Chinese Longitudinal Healthy Longevity Survey (CLHLS). The CLHLS aimed at investigating the determinants of healthy longevity among the older Chinese population aged 65 + and covered 22 of 31 provinces in mainland China [12, 13]. The survey was conducted randomly in about half of the cities/counties in 22 out of 31 provinces in mainland China, covering about 85% of the national population. The surveys investigated socio-economic information, history of health, life habits, and other information by questionnaire and health status by physical examinations. It began in 1998 and continued in 2000, 2002, 2005, 2008, 2011, 2014, and 2018, with about a 90% response rate for each wave [14]. Nearly one-third of participants from each wave were from the previous wave, and the rest were new recruits because of the mixed longitudinal design of CLHLS. To reduce the selection bias in different waves and ensure the consistency of the study population, new recruits were selected based on the similarities in gender, age, and general characteristics with those who were lost during the follow-up. More details of the CLHLS study design can be found elsewhere [12–14].

**Fig. 1:**
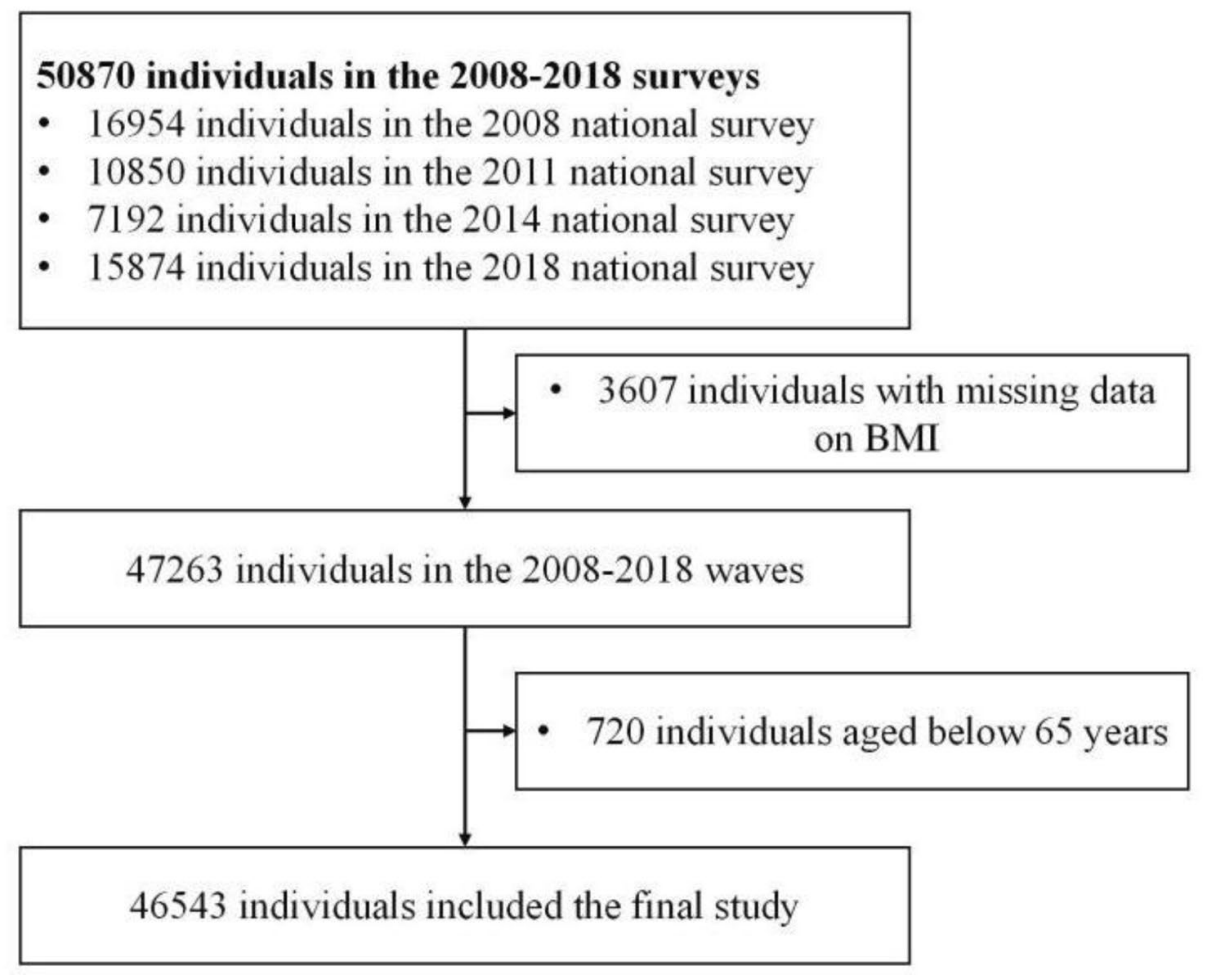
Flowchart of the inclusion of participants. Notes: BMI, Body Mass Index

There was a total of 50,870 participants in the four waves included in our study (16,954 in 2008, 10,850 in 2011, 7,192 in 2014, and 15,874 in 2018). Among them, we excluded 3,607 participants who had missing data on Body Mass Index (BMI) and 720 participants aged below 65 years, yielding 46,543 participants (91.49%) in the final study (Fig. 1). Of which participants aged ≤ 79 years old accounted for 81.93%, 80–89 years old accounted for 16.33%, 90–99 years old accounted for 1.70%, and ≥ 100 years old accounted for 0.04%.

### Body mass index

All information was obtained in the homes of participants through face-to-face questionnaire interviews and physical health examinations by trained investigators. Body weight (in kilograms) and height (in centimeters) were measured by trained assessors following standardized procedures. BMI was calculated by using the unified formula in National Center for Health Statistics as bodyweight (kg) divided by squared body height (m^2^). Participants were divided into four groups according to their BMI using the standard weight status categories from WHO reference, including underweight (BMI < 18.5 kg/m^2^), normal (BMI 18.5–24.9 kg/m^2^), overweight (BMI 25.0-29.9 kg/m^2^), obese (BMI ≥ 30.0 kg/m^2^).

### Explanatory variables

Following previous studies [14–17], we included explanatory variable groups derived from the CLHLS in this study, such as wave indicators (investigation years), demographic factors, socioeconomic status, lifestyle habits, and health conditions variables. Demographic factors included region (urban or rural), gender (male or female), age groups (≤ 79 years,80–89,90–99 or ≥ 100 years), marital status (unmarried, married, or divorced or widowed), and living patterns (living with family members, living in an institution, or living alone). Socioeconomic status included years of schooling (0 years or ≥ 1 years), economic status compared with other local people (poor, fair, or rich). Lifestyle habits included smoking status (never, previous, or current), alcohol intaking status (never, previous, or current), regular exercise (never, previous, or current), dietary diversity (poor, moderate, or good), participating in organized social activities (almost every day, sometimes, or never). Dietary diversity was evaluated as poor (0–3), moderate [4–6], or good [7–9] by the calculated dietary diversity score (0–9) reflecting the consumption numbers of nine types of food groups (meat, vegetables, fish, eggs, fruits, legumes, milk, tea, and nuts) [18]. Health status included body mass index(BMI) (underweight, normal weight, overweight, or obesity), numbers of chronic diseases (0, 1, or ≥ 2), functional disability (no or yes), sleeping quality (good or bad), sleeping length(<5 h, 5–9 h, or ≥ 9 h), self-reported health (good, fair, or poor), self-reported quality of life (good, fair, or poor). Functional disability was defined as the self-reported difficulty with any of the following activities of daily living (ADL) items, such as dressing, eating, bathing, continence, toileting and cleaning, or indoor movement [18].

### Statistical analysis

Baseline characteristics were presented as proportions for categorical variables. Pearson Chi-square tests for trends were used to compare the prevalence rates of abnormal body weights between different baseline characteristics groups, such as survey year, gender, age groups. The multinomial logistic regression model was used to further assess the association between abnormal body weight and potential related factors. In the multinomial logistic regression model, we included survey year, gender, age groups, marital status, residence, economic status, living pattern, education level, numbers of chronic diseases, smoking status, alcohol intaking status, regular exercise, dietary diversity, sleeping quality, sleeping length, housework, outdoor activities, functional disability, self-reported quality of life, self-reported health. In the sensitive analysis, we divided age by per 5 years (65–69, 70–74, 75–79, 80–84, 85–89, ≥ 90 years old) instead of 10 years to examine the robust of the results in the models. A two-tailed P-value of less than 0.05 was considered statistically significant. All the analyses above were performed using Statistical Product and Service Solutions (SPSS 25.0).

## Results

### Characteristics of the study participants

**Fig. 2:**
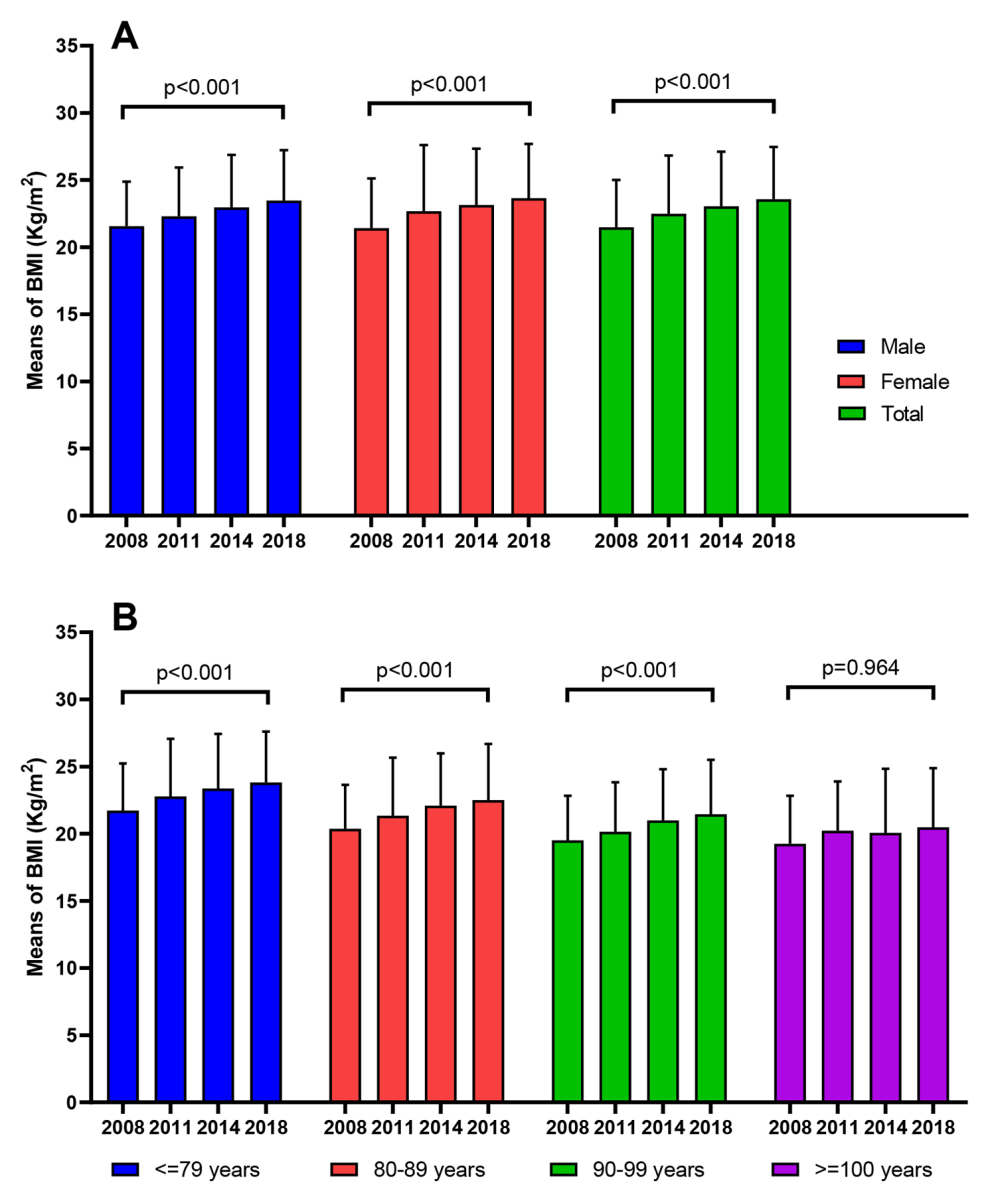
Trends of BMI in different survey years of 2008, 2011, 2014, and 2018. Notes: BMI, Body Mass Index

We included a total of 46,543 participants in four waves of CLHLS surveys in 2008, 2011, 2014 and 2018 (supplemental Table 1). The mean BMI of all subjects was 22.56 ± 3.98 kg/m^2^, with 22.51 ± 3.69 kg/m^2^ for males and 22.61 ± 4.23 kg/m^2^ for females. BMI increased over the years from 2008 to 2018 in both men, women and the all subjects (p < 0.001) in Fig. 2A. Significant differences were observed in the distribution of survey year, gender, living area, family economic status, living pattern, education level, number of chronic diseases, smoking, drinking, regular exercise, dietary diversity score, sleep duration, sleep quality, housework, outdoor sports and other factors in different years (all p < 0.001). Except for the ≥ 100 years old group, BMI in other age groups also showed a trend of increasing with the years (p < 0.001) in Fig. 2B.

### Trends of underweight, overweight, and obesity

From 2008 to 2018, the rate of the underweight and normal weight decreased with each passing year, and the rate of overweight was increasing in the older people in China(Fig. 2B, p<0.05).The prevalence rates of overweight and obesity were higher in female than in male (overweight: 20.37% vs. 19.88%; obesity: 4.79% vs. 2.60%, p < 0.05). Furthermore, the prevalence of overweight and obesity decreased with age but increased among older adults residing in urban areas, higher economic levels, non-smokers and non-drinkers, those experiencing more than 2 chronic diseases, and older adults with higher dietary diversity scores, in comparison to their counterparts, respectively (all p < 0.05, as shown in Table 1). The rates of underweight were higher in females compared to males (15.28% vs. 12.25%, p < 0.05). Additionally, underweight rates increased with age and were also higher among divorced or widowed individuals, older adults residing in rural areas, those with poor family conditions, current smokers or drinkers, and those sleeping less than 5 h, in comparison to their counterparts, respectively (all p < 0.05).

**Table 1:** Comparison of characteristics among 46,543 older adults by BMI groups

	Total	Normal	Underweight	Overweight	Obesity	*p* value
Survey year						< 0.001
2008	16,108	10,554(65.52)	3229(20.05)	2065(12.82)	260(1.62)	
2011	9166	5703(62.22)	1370(14.95)	1690(18.44)	403(4.39)	
2014	6390	4013(62.80)	656(10.27)	1383(21.64)	338(5.29)	
2018	14,879	8738(58.73)	1172(7.87)	4232(28.45)	737(4.95)	
Gender						< 0.001
Male	22,518	14,698(65.27)	2757(12.25)	4476(19.88)	586(2.60)	
Female	24,025	14,310(59.56)	3670(15.28)	4894(20.37)	1151(4.79)	
Age groups (years)						< 0.001
≤ 79	38,133	23,797(62.40)	4522(11.86)	8305(21.78)	1510(3.96)	
80–89	7599	4745(62.45)	1653(21.75)	991(13.04)	211(2.77)	
90–99	792	456(57.57)	246(31.03)	73(9.27)	17(2.13)	
≥ 100	19	10(53.30)	7(38.63)	1(5.90)	0(2.17)	
Marital status						< 0.001
Unmarried	591	369(62.51)	95(16.08)	119(20.11)	8(1.30)	
Married	30,498	19,021(62.37)	3581(11.74)	6777(22.22)	1119(3.67)	
Divorced or widowed	15,219	9474(62.25)	2731(17.95)	2421(15.91)	593(3.89)	
Category of residence						< 0.001
Urban (city and town)	21,402	13,128(61.34)	2183(10.20)	5114(23.90)	977(4.56)	
Rural	25,140	15,881(63.17)	4244(16.88)	4256(16.93)	760(3.02)	
Economic status						< 0.001
Rich	7305	4448(60.89)	738(10.10)	1770(24.22)	349(4.78)	
Fair	33,165	20,923(63.09)	4361(13.15)	6700(20.20)	1181(3.56)	
Poor	5875	3513(59.80)	1310(22.30)	861(14.66)	190(3.24)	
Living pattern						< 0.001
Living with family members	38,725	24,122(62.29)	5258(13.58)	7913(20.43)	1432(3.70)	
Living in an institution	577	350(60.63)	89(15.38)	114(19.71)	25(4.28)	
Living alone	6949	4396(63.26)	1043(15.00)	1245(17.91)	266(3.83)	
Education level (years)						< 0.001
0	18,623	11,512(61.82)	3294(17.69)	3121(16.76)	697(3.74)	
≥ 1 years	27,919	17,496(62.67)	3134(11.22)	6250(22.38)	1040(3.73)	
Numbers of chronic diseases						< 0.001
0	15,557	10,559(67.87)	2345(15.08)	2268(14.58)	385(2.48)	
1	14,465	9002(62.23)	2127(14.71)	2825(19.53)	510(3.53)	
≥ 2	15,811	9007(56.97)	1842(11.65)	4141(26.19)	821(5.19)	
Smoking status						< 0.001
Never	28,867	17,546(60.78)	3914(13.56)	6115(21.18)	1293(4.48)	
Previous	6910	4427(64.06)	873(12.63)	1425(20.63)	185(2.68)	
Current	10,416	6851(65.77)	1614(15.49)	1751(16.81)	200(1.92)	
Alcohol intaking status						< 0.001
Never	26,997	16,302(60.38)	3748(13.88)	5718(21.18)	1229(4.55)	
Previous	4315	2822(65.38)	518(12.00)	853(19.77)	123(2.85)	
Current	10,416	6851(65.77)	1614(15.49)	1751(16.81)	200(1.92)	
Regular exercise						< 0.001
Never	24,526	15,525(63.30)	3867(15.77)	4344(17.71)	790(3.22)	
Previous	3219	2021(62.77)	599(18.61)	493(15.33)	106(3.29)	
Current	18,370	11,210(61.03)	1907(10.38)	4433(24.13)	820(4.46)	
Dietary diversity						< 0.001
Poor	12,357	7708(62.37)	2279(18.44)	1964(15.90)	406(3.28)	
Moderate	23,764	14,957(62.94)	3174(13.36)	4764(20.05)	868(3.65)	
Good	10,343	6277(60.69)	973(9.41)	2631(25.44)	462(4.46)	
Sleeping quality						< 0.001
Good	28,303	17,790(62.85)	3495(12.35)	5981(21.13)	1038(3.67)	
Poor	18,240	11,219(61.51)	2932(16.08)	3390(18.58)	700(3.84)	
Sleeping length						< 0.001
< 5 h	2951	1733(58.73)	538(18.24)	578(19.58)	102(3.44)	
5–9 h	36,701	23,006(62.69)	4813(13.12)	7499(20.43)	1382(3.76)	
> 9 h	6680	4137(61.92)	1047(15.67)	1248(18.68)	249(3.73)	
Housework						< 0.001
Almost everyday	30,679	19,069(62.16)	3979(12.97)	6408(20.89)	1223(3.99)	
Sometimes	6556	4283(65.32)	849(12.94)	1237(18.87)	188(2.87)	
Never	9224	5616(60.89)	1590(17.23)	1695(18.38)	323(3.50)	
Outdoor activities						< 0.001
Almost everyday	24,540	15,461(63.00)	2897(11.80)	5344(21.78)	838(3.41)	
Sometimes	14,045	8578(61.08)	1902(13.54)	2915(20.76)	649(4.62)	
Never	7875	4920(62.47)	1623(20.61)	1084(13.77)	248(3.15)	
Functional disability						< 0.001
No	42,506	26,730(62.88)	5733(13.49)	8528(20.06)	1515(3.56)	
Yes	2958	1611(54.47)	595(20.13)	589(19.92)	162(5.48)	
Self-reported quality of life						< 0.001
Good	57,528	17,888(62.19)	3233(11.24)	6478(22.52)	1165(4.05)	
Fair	30,606	9741(63.66)	2486(16.24)	2577(16.84)	499(3.26)	
Poor	4037	1126(55.77)	558(27.66)	271(13.44)	63(3.13)	
Self-reported health						< 0.001
Good	22,492	14,467(64.32)	2454(10.91)	4811(21.39)	760(3.38)	
Fair	16,824	10,476(62.27)	2290(13.61)	3341(19.86)	717(4.26)	
Poor	6771	3812(56.29)	1532(22.62)	1176(17.37)	251(3.71)	

Notes: BMI, Body Mass Index

### Factors associated with underweight

In the multinomial logistic regression model, older adults in China were significantly more likely to be underweight from 2008 to 2014 compared to the participants in 2018, with a decreasing risk though (OR = 2.33 in 2008, 95% CI: 2.13–2.55; OR = 1.81 in 2011, 95% CI: 1.64–2.01; OR = 1.25 in 2014, 95% CI: 1.11–1.41), after adjusted for other factors (as shown in Table 2). Moreover, male participants were less likely to be underweight compared to female (OR = 0.73, 95% CI: 0.67–0.80). Older adults living in urban areas (OR = 0.72, 95% CI: 0.68–0.77), the currently married (OR = 0.86, 95% CI: 0.80–0.93) and those with good sleep quality (OR = 0.86, 95% CI: 0.80–0.92) had a lower chance of being underweight compared to counterparts, respectively. When comparing the good dietary diversity scores, the results showed that the elderly with poor and fair scores were more likely to be underweight (OR = 1.35, 95% CI: 1.22–1.48; OR = 1.16, 95% CI: 1.06–1.26).

**Table 2:** Multinomial logistic regression model ^a^

	Underweight			Overweight			Obesity	
	OR (95% CI)	*p* value		OR (95% CI)	*p* value		OR (95% CI)	*p* value
Survey year								
2008	2.33(2.13–2.55)	< 0.001		0.42(0.39–0.45)	< 0.001		0.33(0.28–0.39)	< 0.001
2011	1.81(1.64–2.01)	< 0.001		0.63(0.58–0.68)	< 0.001		0.93(0.79–1.08)	0.328
2014	1.25(1.11–1.41)	< 0.001		0.79(0.72–0.86)	< 0.001		1.35(1.15–1.58)	< 0.001
2018	1.00			1.00			1.00	
Gender								
Male	0.73(0.67–0.80)	< 0.001		0.88(0.82–0.95)	0.001		0.60(0.51–0.70)	< 0.001
Female	1.00			1.00			1.00	
Age groups (years)								
≤ 79	0.31(0.09–1.02)	0.054		3.08(0.25–37.22)	0.377		2.42(0.05-127.69)	0.663
80–89	0.54(0.16–1.79)	0.314		1.87(0.15–22.64)	0.623		1.44(0.03–75.94)	0.858
90–99	0.84(0.25–2.82)	0.782		1.50(0.12–18.40)	0.752		1.00(0.02–54.88)	> 0.999
≥ 100	1.00			1.00			1.00	
Marital status								
Unmarried	1.24(0.95–1.62)	0.115		1.48(1.15–1.89)	0.002		0.52(0.23–1.20)	0.125
Married	0.86(0.80–0.93)	< 0.001		1.19(1.11–1.28)	< 0.001		0.97(0.84–1.11)	0.650
Divorced or widowed	1.00			1.00			1.00	
Category of residence								
Urban (city and town)	0.72(0.68–0.77)	< 0.001		1.23(1.16–1.30)	< 0.001		1.26(1.12–1.42)	< 0.001
Rural	1.00			1.00			1.00	
Economic status								
Rich	0.80(0.71–0.91)	0.001		1.12(1.00-1.26)	0.052		1.06(0.85–1.32)	0.621
Fair	0.80(0.73–0.87)	< 0.001		1.06(0.96–1.17)	0.261		0.82(0.68–0.99)	0.037
Poor	1.00			1.00			1.00	
Living pattern								
Living with family members	1.25(1.14–1.38)	< 0.001		1.00(0.91–1.09)	0.988		1.16(0.97–1.38)	0.115
Living in an institution	1.45(1.07–1.95)	0.015		0.67(0.50–0.89)	0.006		0.84(0.49–1.44)	0.525
Living alone	1.00			1.00			1.00	
Education level (years)								
0	1.07(1.00-1.15)	0.042		0.88(0.82–0.93)	< 0.001		0.92(0.82–1.05)	0.216
≥ 1	1.00			1.00			1.00	
Numbers of chronic diseases								
0	1.26(1.16–1.36)	< 0.001		0.47(0.43–0.50)	< 0.001		0.45(0.39–0.52)	< 0.001
1	1.28(1.18–1.38)	< 0.001		0.68(0.64–0.73)	< 0.001		0.64(0.56–0.73)	< 0.001
≥ 2	1.00			1.00			1.00	
Smoking status								
Never	0.55(0.48–0.63)	< 0.001		1.18(1.04–1.32)	0.007		1.47(1.12–1.93)	0.006
Previous	0.67(0.59–0.76)	< 0.001		1.11(0.99–1.23)	0.064		1.12(0.86–1.47)	0.400
Current	1.00			1.00			1.00	
Alcohol intaking status								
Never	1.27(1.13–1.44)	< 0.001		1.13(1.02–1.25)	0.023		1.21(0.97–1.52)	0.093
Previous	1.10(0.89–1.37)	0.371		1.02(0.76–1.36)	0.167		1.15(0.65–2.03)	0.118
Current	1.00			1.00			1.00	
Regular exercise								
Never	1.08(1.00-1.16)	0.041		0.87(0.82–0.92)	< 0.001		0.76(0.67–0.86)	< 0.001
Previous	1.18(1.05–1.33)	0.005		0.78(0.69–0.88)	< 0.001		0.79(0.62–1.01)	0.060
Current	1.00			1.00			1.00	
Dietary diversity								
Poor	1.35(1.22–1.48)	< 0.001		0.72(0.66–0.78)	< 0.001		0.70(0.59–0.83)	< 0.001
Moderate	1.16(1.06–1.26)	0.001		0.86(0.80–0.91)	< 0.001		0.87(0.76–0.99)	0.041
Good	1.00			1.00			1.00	
Sleeping quality								
Good	0.86(0.80–0.92)	< 0.001		1.24(1.17–1.32)	< 0.001		1.26(1.11–1.42)	< 0.001
Poor	1.00			1.00			1.00	
Sleeping length								
< 5 h	1.08(0.93–1.24)	0.311		1.08(0.94–1.24)	0.284		0.85(0.64–1.13)	0.272
5–9 h	1.00(0.91–1.09)	0.944		0.96(0.89–1.04)	0.327		0.91(0.77–1.07)	0.260
> 9 h	1.00			1.00			1.00	
Housework								
Almost everyday	0.89(0.82–0.97)	0.010		1.09(1.00-1.18)	0.043		1.09(0.92–1.28)	0.347
Sometimes	0.83(0.75–0.93)	0.001		0.95(0.86–1.04)	0.269		0.79(0.63–0.99)	0.042
Never	1.00			1.00			1.00	
Outdoor activities								
Almost everyday	0.88(0.81–0.96)	0.005		1.09(1.00-1.20)	0.052		1.02(0.84–1.23)	0.880
Sometimes	1.04(0.95–1.14)	0.436		0.97(0.88–1.06)	0.465		1.48(1.22–1.80)	< 0.001
Never	1.00			1.00			1.00	
Functional disability								
No	0.98(0.86–1.11)	0.706		0.79(0.70–0.89)	< 0.001		0.58(0.47–0.71)	< 0.001
Yes	1.00			1.00			1.00	
Self-reported quality of life								
Good	0.70(0.61–0.80)	< 0.001		1.15(0.98–1.36)	0.088		1.01(0.73–1.39)	0.966
Fair	0.78(0.68–0.88)	< 0.001		0.90(0.77–1.06)	0.201		0.91(0.66–1.24)	0.538
Poor	1.00			1.00			1.00	
Self-reported health								
Good	0.57(0.51–0.62)	< 0.001		1.01(0.92–1.10)	0.881		0.87(0.72–1.04)	0.134
Fair	0.67(0.61–0.73)	< 0.001		0.96(0.88–1.05)	0.353		1.01(0.85–1.19)	0.951
Poor	1.00			1.00			1.00	

Notes: BMI, Body Mass Index; OR, odds ratio; CI, Confidence Interval

^a^ Participates with normal weight as the reference group in the multinomial logistic regression model. In the multinomial logistic regression model, we included survey year, gender, age groups, marital status, residence, economic status, living pattern, education level, numbers of chronic diseases, smoking status, alcohol intaking status, regular exercise, dietary diversity, sleeping quality, sleeping length, housework, outdoor activities, functional disability, self-reported quality of life, self-reported health

### Factors associated with overweight and obesity

Compared to female, male participants were less likely to be overweight or obese (OR = 0.88, 95% CI: 0.82–0.95; OR = 0.60, 95% CI: 0.51–0.70, as shown in Table 2). When comparing the dietary diversity scores and the ADL groups, the results showed that older adults with poor and medium scores (OR = 0.72, 95% CI: 0.66–0.78; OR = 0.86, 95% CI: 0.80–0.91) and those without any functional disability (OR = 0.79, 95% CI: 0.70–0.89) had a lower likelihood of being overweight or obese (poor dietary diversity scores: OR = 0.70, 95% CI: 0.59–0.83; medium dietary diversity scores: OR = 0.87, 95% CI: 0.76–0.99; Independent: OR = 0.58, 95% CI: 0.47–0.71) compared to counterparts, respectively. Compared with the divorced or widowed, the married and the unmarried were more likely to be overweight (OR = 1.19, 95% CI: 1.11–1.28; OR = 1.48, 95% CI: 1.15–1.89). The regression analysis also revealed that older adults living in urban areas (OR = 1.23, 95% CI: 1.16–1.30; OR = 1.26, 95% CI: 1.12–1.42) and with good sleep quality (OR = 1.24, 95% CI: 1.17–1.32; OR = 1.26, 95% CI: 1.11–1.42) had more chances of being overweight or obesity compared to counterparts respectively. The results were similar in the sensitive analysis (supplemental Tables 2–4).

## Discussion

China has the highest number of older adults globally, with approximately 180 million individuals aged 65 years and older, a significant portion of whom suffer from one or more chronic diseases, accounting for 75% of this population [19]. Abnormal body weight is known to be associated with various chronic conditions, leading to significant national healthcare expenditures [20]. We found that obesity prevalence was increasing in all age groups and genders, regardless of geographical location, race or socioeconomic status and especially in the elderly, which was similar to the trends of obesity in other age groups [5, 20]. Our results also show that the prevalence of overweight and obesity increased along with year, which is consistent with other studies [5, 21]. Ampofo et al. [21] utilized prophet models and time-series data from the WHO Global Health Observatory data repository to forecast the prevalence of obesity in 185 countries. They found that obesity prevalence in China was predicted to be 12% in 2030, and called for more urgent attention and effective future interventions.

The obesity prevalence rate found in our study in 2018 (4.95%) was slightly higher than that reported in some Asian countries, such as 4.3% in Japan and 4.7% in Korea [22]. However, it was significantly lower than the rates reported in Western countries, such as 23.1% in the Russian Federation, 28.3% in Canada, 42.8% in the USA, 23.1% in Germany, 20.8% in France, and 20.9% in Spain [22–25]. In the China Chronic Disease and Risk Factors Surveillance program, the prevalence of obesity among adults aged 18–69 years was reported as 8.1%, which was higher than the findings in our study. This difference could be attributed to variations in the age groups targeted in the two national studies. Additionally, the rates of obesity varied widely across different regions and countries, likely influenced by environmental factors that promote obesity. Factors such as improved living conditions, which may lead to improved nutrition and increased calorie intake, the presence of chronic conditions like arthritis that can limit mobility, and the popularity of indoor recreational activities that reduce outdoor physical activities, among others, contribute to this variation [5, 26, 27]. In our results, the rates of overweight and obesity increase among older adults living in the urban areas, rich families, who never smoke or drink, suffering from more than 2 chronic diseases and older adults with good dietary diversity score compared to their counterparts respectively. Our result is consistent with the possible reasons proposed above. In the regression models, we found that adults with poor and medium dietary diversity scores and those without any functional disability had a lower likelihood of being overweight or obese compared to counterpart. Dietary and physical activity has been well-reported to be factors related with obesity. Interestingly, we found that older adults with good sleep quality had higher risk of being overweight and obese. One possible explanation is that older adults with good sleep quality might spend more time in bed and lead to reduced physical activity levels and lower energy expenditures, which might contribute to weight gain over time. Further research is needed to fully understand the mechanisms behind the association between sleep quality and the risk of overweight and obesity among older adults.

For the problem of obesity of the older adults, except for the possible macro sociological and demographic factors, the core biological factors are also significant for effective interventions. Many studies have demonstrated the age-related changes in metabolism and body composition and the presence of chronic disease that develop with the ageing process are the core biological mechanisms and targets [28–30]. In sum, the developing trend of abnormal body weight in older adults is a multifactorial problem with complex interactions among biological mechanisms, sociocultural, environmental factors. There is significant heterogeneity of phenotypes among overweight and obese individuals such as visceral or central obesity, peripheral obesity or metabolically healthy obesity [31]. It limited to classify abnormal body weight only based on BMI value, which may hinder the early identification of metabolic abnormalities. The European Association for obesity research (EASO) stressed that there need to be an improvement in the diagnostic criteria of obesity based on the etiology, degree and health risk [32]. Hopefully, there are more markers or indicators that could help in the subgroup’s classification such as VAT, WHtR and fat mass, or better detection technology for assessment of body composition like dual-energy X-ray [26, 33, 34]. Important progress has been made in identifying subtypes of obesity and what is important is to apply these advances to health management of the older adults.

Compared with most western countries faced with medical burden of obesity, many low-income and middle-income countries are now facing a double burden of disease on underweight, overweight and obesity, especially in Asian countries [35, 36]. Recent evidence has come to a new concept of “obesity paradox” based on the U- or J-shaped relationship between mortality and BMI, which means increased risk of excess mortality were observed in the underweight/normal weight population [37, 38]. Although there has been a slight decrease in the prevalence of underweight, it was estimated that 462 million adults are underweight in 2019[39]. Similarly, in our results, the prevalence rate of the underweight decreased with each passing year from 2008 to 2018 with lowest rate of 7.87% in 2018. Then a multinomial logistic regression model was further performed to explore the influencing factors associated with the underweight. The results show that older adults living in urban areas, married and those with good sleep quality had a lower chance of being underweight compared to counterparts, respectively. Based on our results and other studies, the reasons lying behind the facts can be summarized into two aspects: inadequate food supply and reduced intake [40]. Economic disadvantage, reduced capacity of purchase, psychosocial factors caused by widowhood, divorce, the loss of identity and loss of social roles can result in inadequate food supply [41]. And reduced intake can be result of dementia, polypharmacy, constipation, poor appetite or reduced masticatory efficiency caused by oral health problems [42]. Though being underweight increases the risk of death, dementia, fractures, insulin resistance and higher hospitalization or emergency room visits, less attention appears to be paid to the underweight compared with the overweight and obesity [43–46]. Thus, improvements are needed in public infrastructure and social support, and interventions on nutrition should be given to underweight older adults for healthy aging.

There are several limitations to this study. First, most information like living patterns, lifestyle habits and health status including sleeping quality etc. were collected through face-to-face questionnaire interviews, which may lead to biases in the process of data collection. Moreover, we did not calculate the weighted prevalence of overweight and obesity because of the unavailable survey weights. Second, though this study covered older adults in 22 of 31 provinces in mainland China, limiting the generalization of our results to the geographically or ethnically distinct groups or younger population. Third, based on the cross-sectional study design, our results could not infer the causal relationship between research factors and abnormal weight among older adults.

## Conclusions

In conclusion, the prevalence of overweight/obesity was increasing while the prevalence of underweight and normal weight significantly decreased from 2008 to 2018 among older adults in China after adjustment for demographic confounding factors. This study provided basic information for understanding the changes in body weight and the risk factors of overweight/obesity and underweight in the elderly in China. Future research on abnormal body weight of the elderly in China should consider the use of better detection technology for assessment of body composition and the subgroups classification, fully considering the impact of psychosocial and socioeconomic factors. With the rapidly increasing aging population, the growing overweight/obese older population in China poses a huge challenge for chronic disease and our study directly support intervention policy planning and prevention to the preparation of an aging society.

## Electronic supplementary material

Below is the link to the electronic supplementary material.

Supplementary Material 1

## Data Availability

The datasets generated and/or analysed during the current study are available in the https://opendata.pku.edu.cn/dataverse/CHADS;jsessionid=f69ff64e099fa5139e1708177eec.
