# Supplementary material for: Trends of underweight, overweight, and obesity among older adults in China from 2008 to 2018: a national observational survey
Source: BMC Public Health. 2023 Jul 18;23:1373. doi: 10.1186/s12889-023-16310-6 (PMC10353183; doi:10.1186/s12889-023-16310-6)
Supplement: Supplementary file 1 — Supplementary Material 1 [file 12889_2023_16310_MOESM1_ESM.docx]

**Supplemental files**

**Supplementary table 1. Characteristics of the study participants by survey year**

|  | **2008** | **2011** | **2014** | **2018** | **Total** | ***p* value** |
| --- | --- | --- | --- | --- | --- | --- |
| Gender | | | | | | 0.063 |
| Male | 7711(47.87) | 4526(49.38) | 3135(49.06) | 7145(48.03) | 22518(48.38) |  |
| Female | 8397(52.13) | 4639(50.62) | 3255(50.94) | 7733(51.97) | 24025(51.62) |  |
| Age groups (years) | | | | | | <0.001 |
| ≤79 | 13459(83.55) | 7504(81.87) | 4961(77.62) | 12210(82.07) | 38133(81.93) |  |
| 80-89 | 2426(15.06) | 1514(16.52) | 1275(19.96) | 2383(16.02) | 7599(16.33) |  |
| 90-99 | 216(1.34) | 144(1.58) | 151(2.36) | 281(1.89) | 792(1.70) |  |
| ≥100 | 8(0.05) | 3(0.03) | 4(0.06) | 5(0.03) | 19(0.04) |  |
| Marital status | | | | | | <0.001 |
| Unmarried | 168(1.04) | 168(1.84) | 102(1.61) | 152(1.03) | 590(1.27) |  |
| Married | 10100(62.70) | 5862(64.10) | 4043(64.05) | 10493(71.19) | 30498(65.86) |  |
| Divorced or widowed | 5841(36.26) | 3115(34.06) | 2168(34.34) | 4095(27.78) | 15219(32.87) |  |
| Category of residence | | | | | | <0.001 |
| Urban (city and town) | 6915(42.93) | 4171(45.51) | 2775(43.42) | 7541(50.69) | 21402(45.98) |  |
| Rural | 9193(57.07) | 4995(54.49) | 3616(56.58) | 7337(49.31) | 25140(54.02) |  |
| Economic status | | | | | | <0.001 |
| Rich | 2079(12.93) | 1419(15.53) | 1046(16.46) | 2760(18.69) | 7305(15.76) |  |
| Fair | 11465(71.28) | 6476(70.87) | 4721(74.27) | 10504(71.13) | 33165(71.56) |  |
| Poor | 2540(15.79) | 1243(13.60) | 589(9.27) | 1502(10.17) | 5875(12.68) |  |
| Living pattern | | | | | | <0.001 |
| Living with family members | 13626(84.59) | 7534(82.74) | 5095(80.27) | 12470(84.89) | 38725(83.73) |  |
| Living in an institution | 121(0.75) | 79(0.87) | 145(2.29) | 231(1.58) | 577(1.25) |  |
| Living alone | 2361(14.66) | 1493(16.40) | 1107(17.44) | 1988(13.53) | 6949(15.03) |  |
| Education level (years) | | | | | | <0.001 |
| 0 | 6904(42.86) | 3642(39.74) | 2504(39.18) | 5573(37.46) | 18623(40.01) |  |
| ≥1 | 9204(57.14) | 5523(60.26) | 3887(60.82) | 9305(62.54) | 27919(59.99) |  |
| Numbers of chronic diseases | | | | | | <0.001 |
| 0 | 6073(37.70) | 3143(34.72) | 2179(35.41) | 4163(28.67) | 15557(33.94) |  |
| 1 | 5166(32.07) | 2721(30.07) | 1970(32.01) | 4607(31.73) | 14465(31.56) |  |
| ≥2 | 4870(30.23) | 3187(35.21) | 2006(32.59) | 5749(39.60) | 15811(34.50) |  |
| Smoking status | | | | | | <0.001 |
| Never | 9581(59.51) | 5226(57.39) | 4349(68.34) | 9711(66.40) | 28867(62.49) |  |
| Previous | 2686(16.68) | 1491(16.37) | 695(10.92) | 2039(13.94) | 6910(14.96) |  |
| Current | 3832(23.81) | 2389(26.24) | 1320(20.74) | 2875(19.66) | 10416(22.55) |  |
| Alcohol intaking status | | | | | | <0.001 |
| Never | 9035(61.97) | 4844(58.66) | 4015(70.25) | 9103(69.09) | 26997(64.70) |  |
| Previous | 1712(11.74) | 1025(12.41) | 380(6.65) | 1198(9.10) | 4315(10.34) |  |
| Current | 3832(26.29) | 2389(28.93) | 1320(23.10) | 2875(21.82) | 10416(24.96) |  |
| Regular exercise | | | | | | <0.001 |
| Never | 7982(49.59) | 4315(47.49) | 4130(65.75) | 8100(55.27) | 24526(53.18) |  |
| Previous | 1635(10.16) | 855(9.41) | 173(2.75) | 557(3.80) | 3219(6.98) |  |
| Current | 6477(40.25) | 3917(43.11) | 1978(31.50) | 5998(40.93) | 18370(39.84) |  |
| Dietary diversity | | | | | | <0.001 |
| Poor | 3830(23.78) | 2649(29.03) | 1605(25.13) | 4273(28.78) | 12357(26.59) |  |
| Moderate | 8219(51.02) | 4603(50.45) | 3594(56.28) | 7348(49.49) | 23764(51.14) |  |
| Good | 4060(25.20) | 1872(20.51) | 1187(18.59) | 3225(21.72) | 10343(22.26) |  |
| Sleeping quality | | | | | | <0.001 |
| Good | 10513(65.26) | 5867(64.02) | 4049(63.36) | 7873(52.92) | 28303(60.81) |  |
| Poor | 5595(34.74) | 3298(35.98) | 2341(36.64) | 7005(47.08) | 18240(39.19) |  |
| Sleeping length | | | | | | <0.001 |
| <5 hours | 853(5.30) | 563(6.16) | 412(6.51) | 1123(7.60) | 2951(6.37) |  |
| 5-9 hours | 12629(78.52) | 7089(77.55) | 4992(78.94) | 11991(81.10) | 36701(79.21) |  |
| >9 hours | 2601(16.17) | 1488(16.28) | 920(14.55) | 1671(11.30) | 6680(14.42) |  |
| Housework | | | | | | <0.001 |
| Almost everyday | 10685(66.33) | 6038(65.96) | 4136(64.90) | 9820(66.24) | 30679(66.03) |  |
| Sometimes | 2535(15.74) | 1180(12.89) | 863(13.55) | 1978(13.35) | 6556(14.11) |  |
| Never | 2888(17.93) | 1936(21.15) | 1373(21.55) | 3027(20.42) | 9224(19.85) |  |
| Outdoor activities | | | | | | <0.001 |
| Almost everyday | 9013(55.95) | 5112(55.84) | 3131(49.14) | 7284(49.13) | 24540(52.82) |  |
| Sometimes | 3426(21.27) | 1783(19.47) | 1420(22.29) | 7416(50.02) | 14045(30.23) |  |
| Never | 3669(22.78) | 2260(24.69) | 1821(28.58) | 125(0.85) | 7875(16.95) |  |
| Functional disability | | | | | | <0.001 |
| No | 15341(95.24) | 8121(90.66) | 5570(90.77) | 13474(94.47) | 42506(93.49) |  |
| Yes | 767(4.76) | 836(9.34) | 566(9.23) | 788(5.53) | 2958(6.51) |  |
| Self-reported quality of life | | | | | | <0.001 |
| Good | 8931(56.33) | 5534(60.91) | 4220(66.79) | 10079(67.97) | 28764(62.41) |  |
| Fair | 5942(37.48) | 3117(34.31) | 1921(30.41) | 4322(29.15) | 15303(33.21) |  |
| Poor | 982(6.19) | 434(4.78) | 177(2.81) | 426(2.87) | 2019(4.38) |  |
| Self-reported health | | | | | | <0.001 |
| Good | 7998(50.45) | 4199(46.22) | 3070(48.58) | 7223(48.71) | 22491(48.80) |  |
| Fair | 5285(33.34) | 3358(36.97) | 2469(39.07) | 5711(38.51) | 16824(36.50) |  |
| Poor | 2570(16.21) | 1527(16.81) | 780(12.35) | 1894(12.77) | 6772(14.69) |  |

Notes: BMI, body mass index.

| **Supplementary table 2. Distributions of age among the study participants by survey year** | | | | | | |
| --- | --- | --- | --- | --- | --- | --- |
|  | **2008** | **2011** | **2014** | **2018** | **Total** | ***p* value** |
| Age groups (years) | | | | | | <0.001 |
| 65-69 | 5571(34.59) | 3010(32.84) | 1722(26.94) | 5946(39.96) | 16248(34.91) |  |
| 70-74 | 4675(29.02) | 2569(28.03) | 1868(29.23) | 3820(25.67) | 12931(27.78) |  |
| 75-79 | 3213(19.94) | 1925(21.01) | 1371(21.45) | 2445(16.43) | 8954(19.24) |  |
| 80-84 | 1739(10.80) | 1074(11.72) | 894(13.99) | 1622(10.90) | 5330(11.45) |  |
| 85-89 | 687(4.27) | 440(4.80) | 381(5.97) | 761(5.11) | 2269(4.87) |  |
| ≥90 | 223(1.39) | 147(1.61) | 155(2.42) | 285(1.92) | 811(1.74) |  |

| **Supplementary table 3. Distributions of age among 46543 older adults by BMI groups** | | | | | | |
| --- | --- | --- | --- | --- | --- | --- |
|  | **Total** | **Normal** | **Underweight** | **Overweight** | **Obesity** | ***p* value** |
| Age groups (years) | | | | | | <0.001 |
| 65-69 | 16248 | 9911(61.00) | 1445(8.89) | 4176(25.70) | 716(4.41) |  |
| 70-74 | 12931 | 8208(63.47) | 1651(12.77) | 2601(20.11) | 472(3.65) |  |
| 75-79 | 8954 | 5678(63.42) | 1426(15.92) | 1528(17.07) | 322(3.59) |  |
| 80-84 | 5330 | 3361(63.07) | 1097(20.59) | 728(13.65) | 144(2.70) |  |
| 85-89 | 2269 | 1384(60.99) | 555(24.48) | 263(11.59) | 67(2.94) |  |
| ≥90 | 811 | 466(57.47) | 253(31.21) | 74(9.19) | 17(2.13) |  |

| **Supplementary table 4. Multinomial logistic regression model ^a^** | | | | | | | | |
| --- | --- | --- | --- | --- | --- | --- | --- | --- |
|  | **Underweight** | |  | **Overweight** | |  | **Obesity** | |
|  | **OR (95% CI)** | ***p* value** |  | **OR (95% CI)** | ***p* value** |  | **OR (95% CI)** | ***p* value** |
| Age groups (years) | | | | | | | | |
| 65-69 | 0.27(0.22-0.33) | <0.001 |  | 2.81(2.10-3.76) | <0.001 |  | 3.06(1.70-5.49) | <0.001 |
| 70-74 | 0.35(0.29-0.43) | <0.001 |  | 2.01(1.50-2.68) | <0.001 |  | 2.16(1.20-3.87) | 0.010 |
| 75-79 | 0.44(0.36-0.53) | <0.001 |  | 1.65(1.23-2.21) | 0.001 |  | 2.23(1.24-4.00) | 0.007 |
| 80-84 | 0.59(0.48-0.72) | <0.001 |  | 1.33(0.99-1.78) | 0.063 |  | 1.43(0.79-2.61) | 0.239 |
| 85-89 | 0.71(0.57-0.88) | 0.002 |  | 1.21(0.88-1.67) | 0.233 |  | 1.50(0.80-2.82) | 0.204 |
| ≥90 | 1.00 |  |  | 1.00 |  |  | 1.00 |  |

^a^ Participates with normal weight as the reference group in the multinomial logistic regression model. In the multinomial logistic regression model, we included survey year, gender, age groups, marital status, residence, economic status, living pattern, education level, numbers of chronic diseases, smoking status, alcohol intaking status, regular exercise, dietary diversity, sleeping quality, sleeping length, housework, outdoor activities, functional disability, self-reported quality of life, self-reported health.

OR, Odds ratio; CI, confidence interval.
